# Impact of Top-Down Regulation on the Growth Efficiency of Freshwater Bacterioplankton

**DOI:** 10.3390/microorganisms12102061

**Published:** 2024-10-15

**Authors:** Angia Sriram Pradeep Ram, Hermine Billard, Fanny Perriere, Olivier Voldoire, Jonathan Colombet

**Affiliations:** 1 Laboratoire Microorganismes: Génome et Environnement, UMR CNRS 6023, Université Clermont-Auvergne, 63000 Clermont-Ferrand, France; hermine.billard@uca.fr (H.B.); fanny.perriere@uca.fr (F.P.); jonathan.colombet@uca.fr (J.C.); 2 GEOLAB, UMR CNRS 6042, Université Clermont-Auvergne, 63000 Clermont-Ferrand, France; olivier.voldoire@uca.fr

**Keywords:** bacteria, viruses, heterotrophic nanoflagellates, bacterial mortality, bacterial growth efficiency, freshwater lake

## Abstract

To investigate the hypothesis of top-down control by viruses and heterotrophic nanoflagellates on bacterial-mediated carbon fluxes in freshwater systems, a year-long study (2023–2024) was conducted in the pelagic zone of Lake Saint-Gervais (France). The variability in BGE (9.9% to 45.5%) was attributed to the decoupling of production and respiration, providing bacterioplankton communities with a competitive advantage in adapting to fluctuating environmental disturbances in freshwater systems. The high nucleic acid (HNA) bacterial community, the active fraction, contributed the most to bacterial production and was linked to BGE estimates. Weak bottom-up controls (nutrient concentrations and stoichiometry) on BGE suggested a stronger role for mortality forces. Among viral subgroups (VLP1–VLP4) identified via flow cytometry, the dominant low-fluorescence DNA VLP1 subgroup (range = 0.7 to 3.1 × 10^8^ VLP mL^−1^) accounting for the majority of viral production was closely linked to the HNA population. Both top-down forces exerted antagonistic effects on BGE at the community level. The preferential lysis and grazing of the susceptible HNA population, which stimulated bacterial community respiration more than production in the non-target population, resulted in reduced BGE. These results underscore the key role of top-down processes in shaping carbon flux through bacterioplankton in this freshwater system.

## 1. Introduction

In freshwater systems, complex environmental conditions create unique habitats that support a phylogenetically and metabolically diverse group of heterotrophic prokaryotes [1,2,3]. In surface freshwater, these heterotrophic prokaryotes, primarily bacteria, typically have an average density of 10^6^–10^7^ cells per milliliter. Bacteria play a crucial role in biogeochemical cycles, including organic matter degradation, nutrient cycling, energy transfer, and CO_2_ emissions [4]. In lakes, the input of organic matter, whether from terrestrial sources or through local biological processes, enhances bacterial production (BP) and respiration (BR) by supplying carbon and energy necessary for their metabolic functions [5]. BP reflects the synthesis of new biomass available to higher trophic levels, whereas BR involves the remineralization of organic carbon to CO_2_. The balance between these two processes, known as bacterial growth efficiency (BGE), determines how effectively available substrates support BP and their potential role in heterotrophic food chains [6].

In freshwater systems, the high variability in bacterial growth efficiency (BGE) estimates (ranging from 5% to 80%) over time and space has been linked to the adaptability and plasticity of bacterial communities in response to environmental changes [7]. Numerous studies across various climatic zones have shown that BGE is often constrained by the relative availability of mineral nutrients and organic carbon, which serve as bottom-up resources [8,9]. However, the weak or absent correlation between these resources and bacterial biomass or activity suggests that top-down controls, such as mortality induced by viruses and protistan grazers, are also significant [10]. The extremely high abundance of viruses (10^8^ mL^−1^) in lakes, coupled with their virulent lytic infections, which can account for up to 60% of daily total bacterial mortality [11], underscores their potential role as a regulatory mechanism influencing carbon flux through bacterial communities [12,13]. We hypothesize that viruses, through their host-specific lysis, can significantly impact patterns of BGE. Despite the importance of this relationship, few quantitative studies have examined the variable impact of viruses on BGE over time [14,15,16,17], leaving many questions unanswered, particularly in freshwater systems that are rapidly altered by climate change and human activities.

While our primary focus was on examining the impact of viruses on BGE, it is important to recognize that protistan grazing, mediated by their alterations in dissolved organic matter, can also significantly affect bacterial BGE and influence the flux of organic carbon [18]. Although both viruses and protists contribute to significant bacterial mortality, their impacts on BGE can differ—either positively or negatively—depending on factors such as seasonality, host availability, and environmental conditions. The fact that different members of bacterial community process organic matter with varying efficiencies adds further complexity, as selective lysis or removal of susceptible hosts can lead to the loss of key or limiting nutrients, thereby complicating our understanding of top-down control on BGE. Despite the recognized importance of these top-down forces in freshwater ecosystems, their specific role in carbon sequestration through bacterial carbon metabolism, including both production and respiration, remains insufficiently explored.

Lake Saint-Gervais is an artificial temperate eutrophic lake located in the French Massif Central region, primarily used for recreational purposes and fishing activities. Previous studies have reported a high abundance of viruses [19] and significant lytic infection rates [20] in this lake. Given these findings, it would be valuable to test the hypothesis that viruses, along with flagellates, play a role in controlling bacterial growth efficiency (BGE) by influencing bacterial metabolic parameters. In this study, we evaluated data collected over an annual cycle on cytometrically defined viral clusters and bacterial sub-populations, alongside top-down regulators such as viruses and flagellates, and examined their relationships with bacterial metabolic parameters. This research is part of a broader initiative aimed at understanding the influence of top-down forces on BGE in freshwater ecosystems.

## 2. Materials and Methods

### 2.1. Lake Sampling

Water samples were collected at Lake Saint-Gervais (Supplementary Figure S1, Altitude: 690 m asl; Surface area: 10.5 ha, maximum depth: 4.5 m) over an annual cycle from March 2023 to February 2024. This involved 24 sampling events across four different seasons: spring (March–May), summer (June–August), autumn (September–November), and winter (December–February), which were clearly differentiated by water temperature. During each sampling campaign, 5 liters of lake water were taken from the subsurface (0.5 m) at a designated central pelagic site of the lake (46°02′15′′ N; 2°48′43′′ E) using a PVC Niskin- type water sampler (Bionef, Montreuil, France) during daylight hours. The samples, collected in triplicate during three separate sampling operations, were immediately passed through a 150 µm nylon mesh to exclude larger organisms and then transferred into clean containers. The water samples were stored in refrigerated boxes and transported immediately to the laboratory under cold conditions for further processing.

### 2.2. Physico-Chemical and Chlorophyll Analysis

Water temperature and dissolved oxygen concentration were measured in situ with a submersible probe (YSI Pro DSS, Yellow Springs, OH, USA). For inorganic nutrient analysis, 100 mL of lake water was filtered through a 1.2 µm pore-size polycarbonate filter. The filtrate was then analyzed by a high-pressure ion chromatography technique using Thermo Scientific Dionex System ICS-1100 (Thermo Fischer Scientific, Courtaboeuf, France) for cations (ammonium) and Thermofischer aquion (Thermo Fischer Scientific, Courtaboeuf, France) for anions (nitrite, nitrate, and orthophosphate) [21]. Water color, an indicator of chromophotic dissolved organic matter (CDOM), was measured by reading absorbance at 440 nm on 0.45 µm filtered water samples (2 mL) using a 1 cm quartz cuvette on a spectrophotometer, with Milli Q water as a reference. Color is expressed as a wavelength-specific absorption coefficient in units of inverse meters [22]. Organic carbon measurements for total (water filtered through 150 µm nylon mesh) and dissolved fraction (filtered through 0.7 µm) together with total dissolved nitrogen were determined from each 40 mL water samples using a TOC and nitrogen analyzer (Shimadzu, Kyoto, Japan) [23]. Particulate fraction of organic carbon (POC) was determined from the difference between total (TOC) and dissolved organic fractions (DOC). Chlorophyll *a* concentration (Chl) was determined spectrophotometrically from water samples (200–400 mL) collected on 0.7 μm glass fiber filters (47 mm; GF/F Whatman). Pigments were extracted from the filters with 90% acetone overnight in the dark at 4 °C, and concentrations were calculated from SCOR-UNESCO [24] equations.

### 2.3. Flow Cytometry Counts

Bacteria and viruses were enumerated according to Brussaard et al. [25]. Briefly, 2 mL aliquots of samples were fixed with 37% formaldehyde (EM grade, Sigma, St. Louis, MO, USA) to a final concentration of 1% for 15–30 min at 4 °C. In short, samples were diluted in TE buffer (10 mM Tris HCl, 1 mM EDTA; pH 8.2) and stained with the nucleic acid-specific green fluorescent stain SYBR Green-I (Molecular Probes, Eugene, OR, USA) to a final concentration of 1 × 10^−4^ of the commercial stock at room temperature (15 min) and 80 °C (10 min, after which the samples were cooled at room temperature in the dark for 5 min) for bacteria and viruses, respectively. Samples were enumerated using a FACS Aria Fusion SORP flow cytometer (BD Sciences, San Jose, CA, USA) equipped with an air-cooled Argon laser with an excitation wavelength of 488 nm (15 mW) with a 502 long pass and 530/30 band pass filter set-up. Differences in fluorescence intensity of SYBR-Green I and side scatter signal allowed us to separate bacteria with low nucleic acid content (LNA) from those with high nucleic acid content (HNA) and VLP population into low (VLP1), medium (VLP2), and high fluorescing group (VLP3 and VLP4). All cytometric data were acquired and analyzed with BD FACSDiva 9.0 software (BD Biosciences, San Jose, CA, USA). Control blanks (Tris-EDTA buffer and autoclaved samples) that were processed identically to the samples were checked for very low coincidence and background fluorescence levels prior to sample analysis.

### 2.4. Heterotrophic Nanoflagellate Abundance and Grazing Potential

For enumeration of heterotrophic nanoflagellates (HNFs), triplicate lake water samples were fixed with glutaraldehyde at a final concentration of 2% (EM grade, Sigma Aldrich, St. Louis, MO, USA). The samples (10 to 20 mL) were then stained with Primulin at a final concentration of 20 µg mL^−1^ and incubated in the dark for 15 min [26]. Following staining, the samples were filtered under a vacuum pressure of less than 15 kPa onto 0.8 µm pore size black polycarbonate filters (25 mm, Whatman, Maidstone, UK) with a 1.2 µm backing filter. The filters were then transferred to microscope slides, which were stored at −20 °C in the dark for approximately one week. The samples were subsequently analyzed at ×1000 magnification under UV excitation using a Zeiss Axiovert 200M fluorescence microscope (Göttingen, Germany). For each slide, at least 20 microscopic fields or 200 HNF cells were counted. The loss of bacteria due to HNF grazing was estimated based on in situ bacterial abundance, total HNF abundance, and an assumed mean clearance rate of 2.1 nL HNF^−1^ h^−1^, derived from published reports on freshwater lakes in the French Massif Central [27,28].

### 2.5. Bacterial Production, Respiration, and Growth Efficiency

To determine bacterial growth efficiency (BGE) from bacterial production (BP) and bacterial respiration (BR), lake water was filtered through a 47 mm diameter, 1.2 µm polycarbonate membrane filter (Merck Millipore, Cork, Ireland) using a low differential pressure (<50 mm Hg). This size fractionation procedure separated bacterioplankton from larger planktonic components such as phytoplankton and bacterial grazers, which were confirmed by microscopic observation, allowing for BR and BP measurements with minimal interference. Flow cytometry counts indicated that approximately 70–80% of the free-living bacteria passed through the selected pore size filter.

Of the 4 L water sample, one half was passed through a 150 µm nylon mesh and the other half was filtered through 1.2 µm polycarbonate filters to measure community respiration (CR) and bacterial respiration (BR), respectively [29]. For each fraction, eight gravimetrically calibrated 125 mL borosilicate glass bottles were carefully filled with lake water using snipper system to prevent air bubble formation. The water was allowed to overflow and capped with an air-tight glass stopper. Four bottles were fixed at the start of the incubation (initial time, T0) with Winkler’s reagent, and the remaining were incubated in a water bath at an in situ temperature for 24 h (final time, T24) before fixing. Oxygen concentration was determined using the Winkler titration technique with a potentiometric endpoint detection [30]. Differences in oxygen concentration between the mean of the replicate T0 measurements and the mean of the replicate T24 measurements permitted the calculation of respiration assuming a linear decrease over 24 h. The oxygen values were multiplied by 0.375, to convert them into carbon equivalents, using a respiratory quotient of 1 [31].

BP was assessed based on the specific bacterial growth rate in filtered lake water, as described in our previous investigation [17]. These batch cultures were dark-incubated in acid-washed, pre-combusted glass bottles under the same conditions as the BR samples for a 24 h period. BP was calculated by the increase in the number of cells produced (BA24) over time (t) compared to their initial abundance (BA0). The natural log of bacterial cell abundance was plotted using the least-squared regression method to determine an assumed in situ bacterial specific growth rate (μ) [32]. BP (cells L^−1^ d^−1^) was obtained by multiplying µ with the initial BA and then converted to carbon units using a conversion factor of 20 fg C cell^−1^ [33].

BGE is calculated from the ratio of BP divided by the sum of BP and BR (bacterial carbon demand) and expressed in percentage [5].

### 2.6. Viral Production, Viral Mediated Mortality, and Viral Lysis

Viral production was determined by dilution approach [34]. Briefly, at each sampling event, 50 mL of water samples was mixed with 100 mL of virus-free water (0.02 µm pore size prefiltered) lake water. Sub-samples of 1 mL were immediately collected for viral and bacterial counts (T0 samples) using flow cytometry as described earlier. Samples were incubated in darkness at an in situ temperature and subsampled at every 3 h for a further 9 h. Viral production rates were determined from first-order regressions of viral abundance versus time for triplicate incubations. The lysis rate of bacteria was calculated from the ratio between viral production and burst size (i.e., 30 viruses cell^−1^ as determined during the sampling campaign by the transmission electron microscopy approach, Colombet personal communication). Viral mediated mortality (VMM) was calculated by dividing the lysis rate by the number of bacteria in the original sample, as described by Ordulj et al. [35].

### 2.7. Statistical Analyses

Differences in physico-chemical and microbiological parameters between the seasons were tested by one-way ANOVA. Potential relationships among variables were assessed through linear pair-wise correlations (i.e., Pearson’s correlation analysis) and stepwise multiple regressions. All statistical analyses were conducted using Minitab Version 17 (Minitab Inc., State College, PA, USA). Statistical significance was accepted at an alpha value of *p* < 0.05.

## 3. Result

### 3.1. Limnological Characteristics

Environmental characteristics (mean ± SD) of Lake Saint Gervais (LSG) varied across seasons, as summarized in Table 1. The annual variation of surface lake water temperature ranged from 4.0 °C to 26.0 °C, displaying distinct seasonal patterns. The prevalence of high-water temperatures during the summer months, coupled with the potential for further increases due to climate forcing, can degrade the ecological status of such shallow lakes. This, in turn, can negatively affect the lake’s physical, chemical, and biological properties, which are interconnected and influence one another. Dissolved oxygen concentration, ranging from 7.7 to 12.3 mg L^−1^, which corresponded to between 78% and 110% of oxygen saturation levels, indicated no evident deoxygenation at any point. On an annual basis, temperature and dissolved oxygen concentration were inversely correlated (r = −0.61, *p* < 0.001), typical of temperate freshwater systems. Lake conductivity, indicative of saline conditions and water quality, remained consistently below 200 µs/cm, representative of streams and lakes. Nitrate concentrations increased significantly (*p* < 0.001) from the start to the end of the study, with the highest concentrations observed in winter (mean = 684 ± 156 µg L^−l^). In contrast, ammonia and nitrite concentrations did not exhibit significant seasonal trends (Table 1). Ortho-phosphate concentrations were mostly below detectable limits (<40 µg L^−1^), with the highest value of 937 µg L^−1^ recorded in late September. Dissolved organic carbon (DOC), which ranged from 6.4 to 10.1 mg L^−1^ (mean = 8.2 ± 1.2 mg L^−1^), displayed seasonal patterns that aligned with changes in water temperature (Supplementary Figure S2). DOC accounted for approximately 61% to 90% of the total organic carbon fraction (range = 7.7–14.0 mg L^−1^), suggested variable inputs of organic matter, likely available for microbial degradation. Unlike, the DOC, the total dissolved nitrogen showed a high concentration in the winter (mean = 1.3 ± 0.2 mg L^−1^) compared to other seasons (<0.7 mg L^−1^). The annual mean C:N ratio (mean = 10.6 ± 3.2) of dissolved organic matter was much lower than the reported threshold (<15) level for inducing limitations on microbial growth and activity in lake systems. The low water color (<3.0 m^−1^) and its weak relationship with dissolved organic carbon indicated minimal organic matter inputs of humic origin contributing to colored dissolved organic matter pool. Chlorophyll a concentration, an indicator of algal biomass, fluctuated 18-fold between 1.8 and 32.6 µg L^−1^ (mean = 15.0 ± 9.4 µg L^−1^), with significantly lower values (*p* < 0.005) in summer (mean = 6.3 ± 4.2 µg L^−1^) compared to other seasons (>15.1 µg L^−1^). A poor relationship (*p* > 0.05) between chlorophyll and POC together with their high ratio (POC: Chl > 100) indicated the greater presence of detrital sources of organic carbon in LSG.

### 3.2. Abundances of Viruses, Bacteria, and Heterotrophic Nanoflagellates

**Figure 1:**
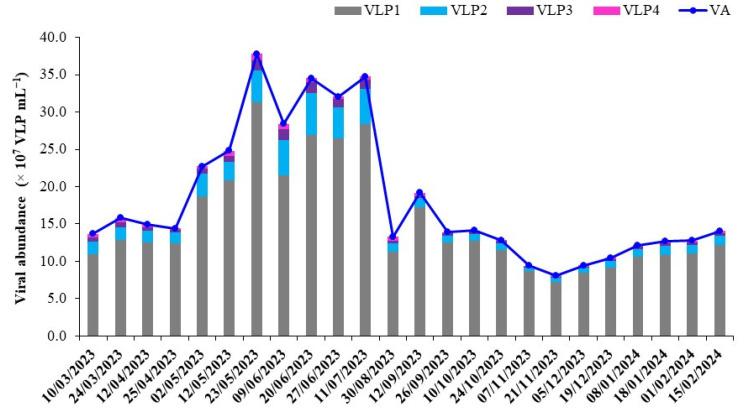
Seasonal variations in total viral abundances (VA), expressed as viral-like particles (VLP), alongside the distribution of viral subgroups (VLP1, VLP2, VLP3, and VLP4) distinguished by their fluorescence characteristics using flow cytometry. The data presented represent the averages of triplicate measurements.

Seasonal variability in microbial standing stocks (viruses, bacteria, and heterotrophic nanoflagellates) and their mediated activity in LSG are summarized in Table 2. The temporal distribution patterns of viral (VA) and bacterial abundances (BA) followed similar trends over a one-year period, correlating with variations in water temperature and dissolved organic carbon concentrations (Table 3). ANOVA revealed marked seasonal differences in viral and bacterial abundances, with higher standing stocks during the warm summer months (mean VA = 27.0 ± 8.9 × 10^7^ VLP mL^−1^, mean BA = 11.4 ± 2.2 × 10^6^ cells mL^−1^) compared to other periods (Figure 1). Within the total viral abundance (VA, expressed as virus-like particles VLP), up to four viral subgroups (VLP1-VLP4) displaying various fluorescence intensities based on DNA content were identified from cytograms. Overall, VA ranged from 8.1 to 37.8 × 10^7^ VLP mL^−1^ (mean = 19.0 ± 9.0 × 10^7^ VLP mL^−1^), with a gradual decline from summer towards the start of autumn until winter. The maximum VA observed in late spring was 4.7 times higher than the lowest value obtained in late November. The low-fluorescent DNA VLP1 group, mainly comprising small viruses, exhibited less seasonal variability than other subgroups and was the most abundant, making up about 76 to 92% (mean = 85.1 ± 4.3%) of the total viral population. VLP1 ranged between 7.2 and 31.3 × 10^7^ VLP mL^−1^ (mean = 18.2 ± 9.0 × 10^7^ VLP mL^−1^) and on average was 3, 12, and 22 times higher than the VLP2, VLP3, and VLP4 groups, respectively.

**Figure 2:**
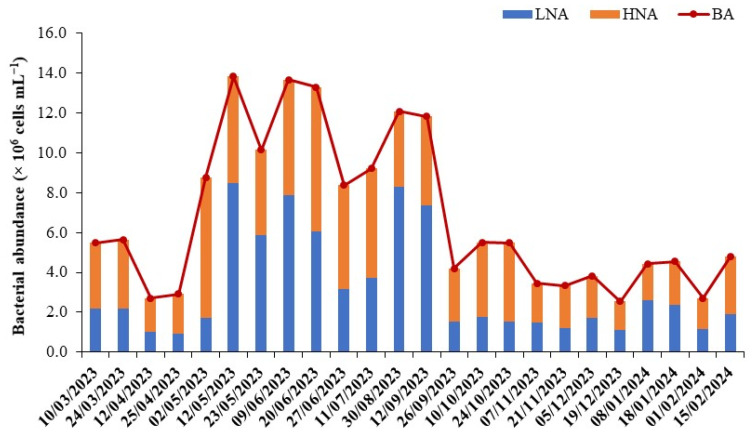
Seasonal variations in total heterotrophic bacteria (BA) and their subgroups, categorized as low nucleic acid (LNA) and high nucleic acid (HNA) bacteria, identified using flow cytometry. The data shown represent the averages of triplicate measurements.

The total heterotrophic bacterial abundance (BA) exhibited seasonal variation, ranging from 2.5 to 13.8 × 10^6^ cells mL^−1^ (mean = 7.1 ± 3.9 × 10^6^ cells mL^−1^). Low values were recorded in winter and high values in summer (Figure 2). The maximum observed in early June was 5.6 times higher than the lowest winter value. Flow cytometry dot plots of green fluorescence against side scatter (SSC) differentiated the bacterial community into two subgroups: low nucleic acid (LNA) and high nucleic acid (HNA) content bacteria. LNA and HNA abundances ranged from 0.9 to 8.5 × 10^6^ cells mL^−1^ and from 1.4 to 7.6 × 10^6^ cells mL^−1^, respectively, with peaks in spring and summer. Environmental conditions such as temperature and dissolved organic carbon facilitated the proliferation of HNA cells, which dominated the LNA community by an average of 1.5-fold. Both subgroups showed similar seasonal trends, but LNA displayed higher variability (coefficient of variation, CV = 77%) compared to the HNA community (CV = 48%).

The virus-to-bacteria ratio (VBR), serving as a proxy for viral activity or the balance between viral decay and production, ranged from 11 to 55, with an overall mean of 29.2 ± 10.4. Both bacterial and viral counts equally contributed to the variability in VBR. No significant seasonal variations in VBR were observed. Heterotrophic nanoflagellate (HNF) abundance ranged from 2.6 to 11.4 × 10^3^ cells mL^−1^, with a mean of 5.7 ± 2.5 × 10^3^ cells mL^−1^, peaking in the autumn season (Table 2). Their distribution pattern was not influenced by any of the measured physico-chemical parameters.

### 3.3. Bacterial Production, Respiration, Carbon Demand, and Growth Efficiency

**Figure 3:**
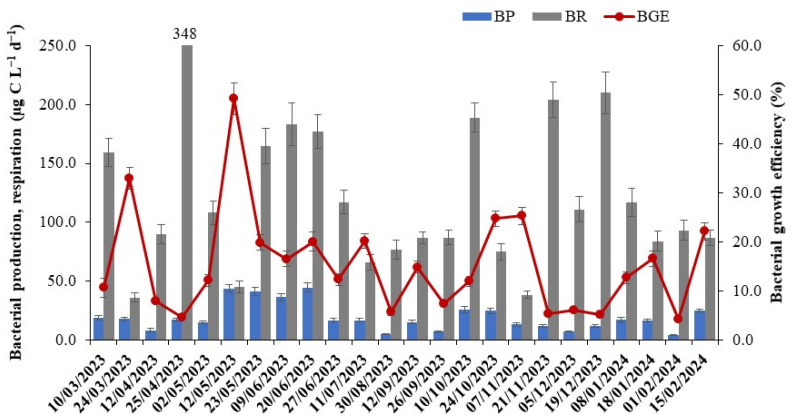
Seasonal dynamics of bacterial metabolic parameters, including bacterial production (BP) and respiration (BR), along with the calculated bacterial growth efficiency (BGE) in the pelagic zone of Lake Saint-Gervais. Error bars represent the standard deviation (SD) from triplicate measurements (*n* = 3).

Overall, bacterial production (BP) varied by an order of magnitude over an annual cycle, ranging between 4.2 and 44.4 µg C L^−1^ d^−1^, with significantly (*p* < 0.02) higher activity in spring–summer compared to autumn and winter (Figure 3). Abiotic parameters such as temperature and dissolved organic carbon influenced BP across the seasons. In contrast, bacterial respiration (BR), which ranged from 36 to 348 µg C L^−1^ d^−1^ (mean = 123.0 ± 70.7 µg C L^−1^ d^−1^), was more conservative than BP and displayed no seasonal variations (Figure 3). BR was significantly higher (*p* < 0.001) than BP by an average of sixfold. Bacterial growth efficiency (BGE), integrating both metabolic parameters (BP and BR), ranged from 4.3% to 49.2% (mean = 15.4 ± 10.5%) (Figure 3). The peaks in BP and BR were found to covary, suggesting that the community adjusts its metabolism between growth and cell maintenance depending on the nutrient environment. Thus, the decoupling between these two bacterial metabolic parameters resulted in the observed variability in BGE (by an order of magnitude) across the study seasons. Bacterial carbon demand (the sum of BP and BR), which varied from 51.4 to 364.9 µg C L^−1^ d^−1^, was not entirely supported by phytoplankton carbon source, suggesting it was likely fueled by inputs of external organic matter. Unlike BP, none of the measured abiotic parameters significantly impacted BR and BGE. BP accounted for 41% of the variability in BGE, leading to a negative relationship (*p* < 0.001) between BR and BGE (Table 3). Overall, BR contributed to 30–93% of the microbial community respiration, reiterating its importance in influencing carbon cycling in freshwaters. Community respiration ranged from 84.0 to 357.2 µg C L^−1^ d^−1^, with maxima coinciding with those of BR.

### 3.4. Viral Production, Lysis, and Potential Flagellate Grazing

Rates of viral production (VP) varied by an order of magnitude across the seasons, ranging from 6.4 to 76.2 × 10^6^ VLP mL^−1^ d^−1^, with significantly higher rates (*p* < 0.03) in spring–summer than in autumn–winter (Table 2). Lysis, calculated from VP and an average burst estimate of 30 by transmission electron microscopy, varied between 2.1 and 25.4 × 10^8^ cells L^−1^ d^−1^ (mean = 9.1 ± 5.1 × 10^8^ cells L^−1^ d^−1^). Among the loss factors, heterotrophic nanoflagellates exhibited dominance over viruses, with grazing rates between 3.7 and 35.4 × 10^8^ cells L^−1^ d^−1^ (mean = 15.2 ± 9.5 × 10^8^ cells L^−1^ d^−1^). Overall, potential flagellate grazing rates were significantly higher (*p* < 0.007) than viral lysis by an average of 1.8-fold. Expressed as a percentage of the bacterial stock, viral lytic infection destroyed about 4.0 to 47.6% of the total bacterial community, with significantly (*p* < 0.02) lower values in summer than in colder months. High lysis coinciding with the lowest bacterial growth efficiency (BGE) estimates was eventually accompanied by maxima in bacterial respiration (BR) in late April.

### 3.5. Relationships between Variables

**Figure 4:**
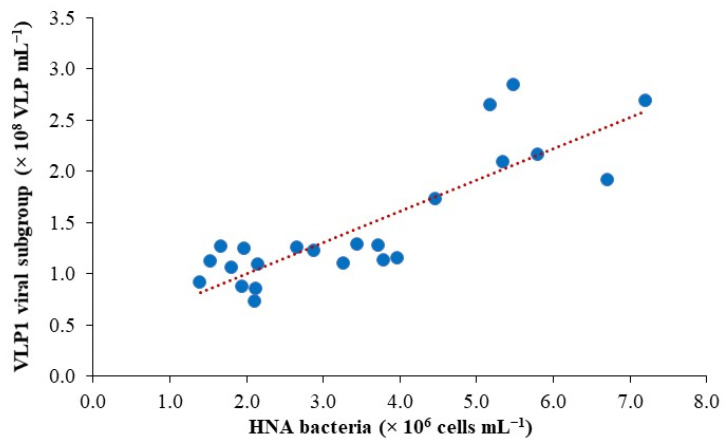
A regression plot showing the relationship between high nucleic acid bacteria (HNA) and the low-fluorescent viral DNA subgroup VLP1 in Lake Saint-Gervais (y = 0.31x + 0.39, r^2^ = 0.73, *p* < 0.001, *n* = 24).

Environmental factors, particularly water temperature and the availability of dissolved organic carbon, had a considerable impact on microbial abundances and their associated activity (Table 3). Overall, viral abundance (VA) was significantly correlated with bacterial abundance (BA) (r = 0.72, *p* < 0.001) rather than with chlorophyll a and phytoplankton abundance. This highlights the importance of the bacterial community serving as principal hosts for viral proliferation in LSG. A strong association (*p* < 0.001) of high nucleic acid (HNA) cell abundance with bacterial production (BP) indicates the active nature of HNA cells within the total bacterial population, suggesting they play a disproportionately large role in the freshwater carbon flux (Supplementary Figure S3). Due to their active nature, the HNA community was preferentially targeted by the low fluorescent VLP1 viral subgroup, which mostly comprises bacteriophages (viruses that infect bacteria). Within the dominant members of the bacterial and viral communities, the HNA fraction was a strong predictor of VLP1, accounting for 70% of the variation: the regression equation (Model II) that relates HNA to VLP1 is as follows: VLP1 = 0.30HNA + 0.42, r^2^ = 0.73, *p* < 0.0001, *n* = 24 (Figure 4).

**Figure 5:**
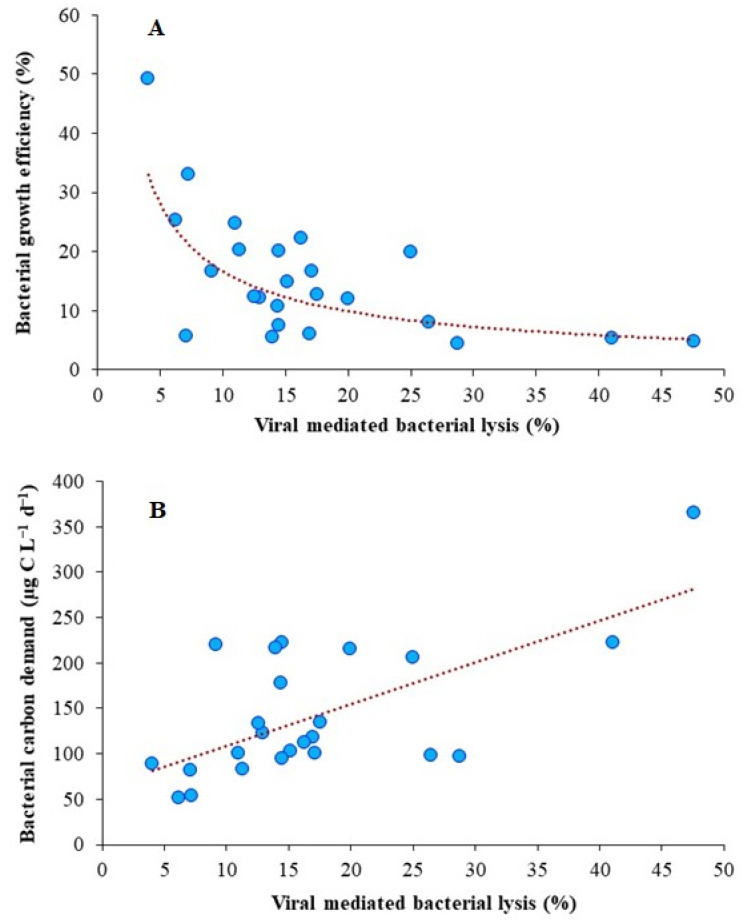
Relationship of viral lysis with bacterial growth efficiency ((**A**), y = 94.35x^−0.75^, r = −0.75, *p* < 0.001, *n* = 24) and carbon demand ((**B**), y = 4.59x + 63.48, r = 0.65, *p* < 0.001, *n* = 24) in Lake Saint-Gervais.

A significant positive correlation (*p* < 0.001) between viral abundance and production supports the argument of selective removal of susceptible members of the bacterial community, which, in our case, was HNA. Unlike HNA grazing, viral lysis was negatively correlated (*p* < 0.001) with bacterial growth efficiency (BGE) through an increase in bacterial carbon demand (*p* < 0.001) (Figure 5A,B). The contribution of HNA to BGE and its subsequent lysis indicates its dynamic nature of this dominant subgroup in contributing to high production and growth. In LSG, changes in total bacterial abundances could be linked to changes in the HNA fraction. Flagellate grazing developed a positive relationship with bacterial abundance (*p* < 0.001) and production (*p* < 0.01), suggesting the importance of grazers in the control of bacterial community. The lack of a relationship between BGE and the measured abiotic parameters, along with nutrient stoichiometry, highlights the greater significance of top-down control over resource control in regulating carbon flux through bacterioplankton.

## 4. Discussion

In this investigation, our findings underscore the significant influence of top-down factors (viruses and heterotrophic nanoflagellates) on bacterial-mediated carbon flux (referred to as bacterial growth efficiency, BGE) in a eutrophic freshwater system, a topic that has been largely underexplored in aquatic environments. The weak association or lack of correlation between bottom-up factors (such as nutrient concentrations and their stoichiometric ratios) and BGE suggested a stronger role for mortality forces. Antagonistic relationship of top-down forces with BGE indicated that these biotic agents operated at the community level, reducing the overall BGE of the non-infected population through selective lysis and grazing of dominant active community members that typically contribute to higher BGE.

### 4.1. Lake Environment

In LSG, the large variability in DOC:TOC ratios suggest a seasonal input of organic matter that could originate from both allochthonous (terrestrial) and autochthonous (phytoplankton) sources. The high POC ratio (mean = 226 ± 184), which exceeded the commonly reported threshold level of 100, along with the poor correlation between POC and Chl, indicated the dominance of detrital or non-phytoplankton material in the system [36]. In small-sized lakes (<5 km^2^), catchment sources and artificial outlets are often the primary contributors of organic matter loading [37]. The in situ nutrient concentrations and the carbon, nitrogen, and phosphorus stoichiometry of the dissolved organic matter were within threshold levels, preventing any limitation of bacterial growth [38]. The environmental conditions in the lake, characterized by high viral (3.8 ± 0.7 × 10^8^ VLP mL^−1^) and bacterial abundances (1.4 ± 0.4 × 10^7^ cells mL^−1^), were strongly influenced by water temperature and dissolved organic carbon, which acted as significant factors driving the observed temporal variability. The negative correlation between bacteria and chlorophyll under high DOC conditions suggests an increased reliance of bacterial activity on detrital sources of carbon, a pattern commonly observed in euphotic systems [39].

Cytometric sorting techniques have identified four taxonomic distinctions among viral sub-groups, paralleling those observed in bacterial subpopulations [40]. In LSG, the low-fluorescence VLP1 community accounted for 80% of the total viral abundance [41,42]. Their temporal distribution was similar to that of larger genome-size subgroups, such as VLP2, VLP3, and VLP4, which are mainly known to infect eukaryotic and algal populations [25,43]. Within the bacterial community, two subgroups, namely high nucleic acid (HNA) and low nucleic acid (LNA) cells, which represent different physiological states, were distinctly identified. The predominance of the environmentally sensitive HNA community over their LNA counterparts in most months can be attributed to system productivity, particularly organic carbon availability [40,44]. HNA cells have been used as bioindicators for assessing anthropogenic organic inputs in freshwater environments [45]. The significant correlation between HNA cells and bacterial production supports the idea that they represent an active fraction with high cell-specific activity, reflecting a proportionally greater flux of carbon through these cells [40,46]. Often classified as r-strategists or opportunistic species, HNA cells possess large and flexible genomes, allowing them to exploit nutrient pulses and occupy diverse ecological niches [47,48].

The temporal variability of viruses in the pelagic realm was primarily influenced by the distribution of their main host, bacteria. Environmental factors that correlated with bacterial abundance also correlated with viral abundance, suggesting that virus dynamics in LSG are linked to host abundance, productivity, and grazing pressure [49]. Among the viral and bacterial subgroups, regression results strongly suggest that the low fluorescence VLP1 cluster, which predominantly comprises bacteriophages, was dependent on the HNA bacterial community. This implies that the in-situ production of viruses relies on the active group of the host community, which contributes to high rates of bacterial production [50]. HNA bacteria explained a substantial variation (70%) in VLP1 abundance over time. Known for their high virulence (short latent period) and large burst size [51], the VLP1 cluster’s ability to rapidly infect the active HNA bacterial population can significantly regulate the flux of carbon through higher trophic levels. In LSG, the highest virus-to-bacteria ratio (VBR) coincided with increased viral production, likely due to the virulent nature of the VLP1 group, which could be responsible for increased bacterioplankton lysis, as observed in other aquatic systems [52].

Despite the high standing stocks of bacteria and viruses, along with increased contact rates, viral lysis appeared to contribute modestly to bacterial mortality. This paradox of low viral infection rates despite high virus–bacteria contact in eutrophic systems could be due to the resistance of certain bacterial subpopulations to co-occurring viruses, shifts in the length of the lytic cycle, or viral decay [15]. Similar to viruses, the HNA bacterial community was also targeted by heterotrophic nanoflagellates (HNFs), likely due to their larger size compared to the relatively smaller LNA cells [53]. When comparing the two sources of bacterial mortality, it was observed that potential flagellate grazing generally exceeded viral lysis in most months. Because HNFs are size-selective, grazing on virus-infected cells could also have contributed to the lower observed estimates of viral lysis [54].

### 4.2. Top-Down Impact on Bacterial Growth Efficiency

Studies on aquatic systems show that BGE depends on the relative availability of mineral nutrients and organic carbon [5,8,29]. In lake ecosystems, BGE fluctuates with changes in nutrient concentrations, with higher values in nutrient-rich periods. Across ecosystems, BGE increases from oligotrophic to eutrophic systems, primarily driven by nutrient availability [31]. In LSG, the uncoupling between metabolic parameters, such as bacterial production and respiration, contributed to the seasonal variability of the calculated BGE, which ranged from 4.3% to 49.2% [55,56]. This phenomenon, commonly observed in lake systems, is likely due to shifts in bacterial metabolism towards cellular maintenance rather than biomass production, allowing bacteria to cope with the fluctuating environmental conditions [57,58]. Additionally, the decoupling between bacterial production and respiration can also result from top-down mediated shifts in the community structure, which affect microbial energetics and activity, thereby influencing patterns in BGE [18,59]. The lack of a significant correlation between BGE and the studied abiotic parameters, including nutrient levels and stoichiometry of organic matter, suggests that substrate quality alone does not sufficiently explain its temporal variability. This indicates the importance of bacterial interactions with other segments of the microbial community, such as viruses and grazers [60]. The significant relationship between viral lysis and bacterial carbon demand in LSG aligns with theoretical models and experimental studies using seawater cultures, where the viral-mediated recycling of bacterial carbon significantly influences the flux of organic carbon in ocean systems [61,62]. Metagenomic analyses have further revealed that viruses can significantly impact bacterial-mediated carbon fluxes in freshwater systems by utilizing auxiliary metabolic genes that modify host metabolism [63].

Viruses had an adverse effect on BGE. This overall decrease in BGE at the community level was due to the preferential lysis of highly active HNA bacteria and a positive relationship between viral lysis and respiration. This relationship can be explained by the high energy expenditure required for the non-targeted bacterial population to process viral lysed organic matter through extracellular enzymatic activity for incorporation into biomass [64]. A study from Lake Biwa indicated that prokaryotic species contributing significantly to total abundance and bulk production were infected and suppressed by viruses through density- and frequency-dependent regulation, driving the viral shunt [65]. The oxidation of organic matter released from bacterial cells via the viral shunt has been shown to result in up to 27% more bacterial respiration and production, leading to a lower flow of organic carbon to higher trophic levels [66,67]. This scenario, where increased bacterial respiration is accompanied by low BGE, resulting in a negative relationship between viral lysis and BGE, has been reported in both marine [68] and freshwater systems [15,42]. Top-down processes induce changes in bacterial communities, which are reflected in their overall bulk metabolism.

Additionally, HNF also negatively impacted BGE, likely through the grazing of large and active HNA cells, as indicated by the significant correlation between HNF abundance and bacterial production. The significant flexibility in the grazing impact of certain bacterivorous flagellate communities has previously been shown to exert complex top-down pressure on dominant bacterial species, potentially contributing to the carbon flow in aquatic systems [69,70]. Previous studies have shown that protist-altered dissolved organic matter can lead to a strong increase in bacterial carbon demand, resulting in decreased BGE due to high bacterial respiration rates [18]. The nature of organic matter released due to viral lysis and grazing can variably impact BGE [71,72], with the relationship largely depending on the nutrient status of the ecosystem. For example, during nutrient-limited conditions, organic matter released by top-down activity has been shown to have positive effects on BGE [17]. Although mortality leads to the release of regenerated nutrients into the environment, the benefit of these resources to non-targeted bacteria is likely minimal in eutrophic systems compared to oligotrophic systems.

Our investigation clearly showed that both viruses and HNF exhibited a preference for the active HNA bacterial community. This combined impact of mortality agents selectively suppressing metabolically active, dominant taxa aligns with the “phage kills the winner” hypothesis [62]. Such selective repression can influence the overall BGE of the entire community, with significant implications for bacterial-mediated carbon cycling in freshwater ecosystems. In LSG, both top-down factors negatively impacted BGE at the community level primarily through their mortality processes rather than bottom-up processes. The findings of our study pave the way for further exploration of the link between bacterial carbon metabolism and community structure, which is critical for ecosystem functioning, as these factors affect nutrient remineralization and carbon flow in aquatic systems. However, changes in bacterial community structure due to top-down effects need to be further assessed in future studies. Given that both resources and mortality forces tend to operate simultaneously and interactively to control bacterial communities and functions, it is important to recognize that the relative roles of these control mechanisms in regulating bacterial metabolism can vary over time within a system, both spatially and temporally, or among lakes with different trophic statuses.

## Figures and Tables

**Table 1:** Physico-chemical characteristics and chlorophyll concentrations (mean ± SD) of Lake Saint-Gervais during the study period (March 2023–February 2024). Values with different superscripted letters are significantly (*p* < 0.05) different.

Parameters	Spring	Summer	Autumn	Winter
Water temperature (°C)	12.8 ± 3.9 ^a^	23.4 ± 2.2 ^b^	13.1 ± 4.3 ^a^	5.6 ± 1.6 ^c^
Dissolved oxygen (mg L^−1^)	10.1 ± 1.5 ^a^	9.1 ± 1.6 ^a^	9.5 ± 0.3 ^a^	9.9 ± 0.7 ^a^
Conductivity (µs cm^−1^)	165.0 ± 7.2 ^a^	168.5 ± 8.5 ^a^	170.0 ± 6.9 ^a^	156.5 ± 0.3 ^b^
Water color (m^−1^)	1.3 ± 0.6 ^a^	1.1 ± 0.7 ^a^	0.6 ± 0.4 ^b^	1.2 ± 0.9 ^a^
Ammonium (µg L^−1^)	38.2 ± 36.5 ^a^	15.2 ± 12.2 ^b^	53.3 ± 51.5 ^c^	88.0 ± 99.4 ^d^
Nitrite (µg L^−1^)	11.2 ± 1.9 ^a^	10.0 ± 1.1 ^a^	14.5 ± 7.7 ^a^	13.1 ± 3.9 ^a^
Nitrate (µg L^−1^)	30.3 ± 9.8 ^a^	87.5 ± 112.5 ^b^	147.7 ± 207.9 ^c^	684.5 ± 155.8 ^d^
Orthophosphate (µg L^−1^)	<40.0 ^a^	<40.0 ^a^	339.1 ± 518 ^b^	56.9 ± 27.2 ^c^
Total organic carbon (mg L^−1^)	11.0 ± 1.3 ^a^	12.1 ± 1.7 ^a^	10.2 ± 1.3 ^a^	8.4 ± 0.4 ^b^
Dissolved organic carbon (mg L^−1^)	8.6 ± 0.3 ^a^	9.4 ± 0.6 ^a^	7.9 ± 0.9 ^a^	6.6 ± 0.1 ^b^
Particulate organic carbon (mg L^−1^)	2.4 ± 1.5 ^a^	2.7 ± 1.7 ^a^	2.3 ± 0.8 ^a^	1.8 ± 0.4 ^b^
Total dissolved nitrogen (mg L^−1^)	0.7 ± 0.6 ^a^	0.7 ± 0.1 ^a^	0.7 ± 0.1 ^a^	1.2 ± 0.3 ^b^
C:N ratio	11.9 ± 0.7 ^a^	12.9 ± 1.5 ^a^	11.9 ± 1.5 ^a^	5.8 ± 1.5 ^b^
Chlorophyll a (µg L^−1^)	15.2 ± 8.0 ^a^	6.3 ± 4.2 ^b^	19.3 ± 7.3 ^c^	19.9 ± 11.5 ^c^

**Table 2:** Microbial characteristics (mean ± SD) of Lake Saint-Gervais during the study period (March 2023–February 2024). Values with different superscripted letters are significantly (*p* < 0.05) different.

Parameters	Spring	Summer	Autumn	Winter
Total viral abundance (10^7^ VLP mL^−1^)	20.6 ± 8.8 ^a^	27.0 ± 8.9 ^a^	11.7 ± 3.8 ^b^	12.0 ± 1.7 ^b^
VLP1 viral subgroup (10^7^ VLP mL^−1^)	17.1 ± 7.3 ^a^	22.0 ± 6.7 ^b^	10.5 ± 2.5 ^c^	10.4 ± 1.4 ^c^
VLP2 viral subgroup (10^7^ VLP mL^−1^)	2.3 ± 1.0 ^a^	3.6 ± 1.9 ^b^	0.8 ± 0.2 ^c^	1.0 ± 0.3 ^c^
VLP3 viral subgroup (10^7^ VLP mL^−1^)	0.7 ± 0.4 ^a^	1.0 ± 0.5 ^a^	0.2 ± 0.08 ^b^	0.3 ± 0.08 ^b^
VLP4 viral subgroup (10^7^ VLP mL^−1^)	0.5 ± 0.2 ^a^	0.5 ± 0.1 ^a^	0.1 ± 0.02 ^b^	0.2 ± 0.05 ^b^
Total bacterial abundance (10^6^ cells mL^−1^)	7.1 ± 4.1 ^a^	11.4 ± 0.2 ^b^	4.4 ± 1.1 ^c^	3.8 ± 0.9 ^c^
HNA abundance (10^6^ cells mL^−1^)	3.9 ± 1.9 ^a^	5.3 ± 1.2 ^b^	2.9 ± 0.9 ^c^	2.0 ± 0.5 ^c^
LNA abundance (10^6^ cells mL^−1^)	3.2 ± 2.9 ^a^	6.1 ± 2.2 ^b^	1.5 ± 0.2 ^c^	1.8 ± 0.6 ^c^
Virus to bacteria ratio	34.1 ± 13.7 ^a^	25.0 ± 11.2 ^b^	26.7 ± 3.8 ^b^	33.1 ± 9.1 ^a^
Bacterial production (µg C L^−1^ d^−1^)	23.0 ± 13.7 ^a^	22.3 ± 14.9 ^a^	16.4 ± 8.3 ^b^	13.5 ± 7.5 ^b^
Bacterial respiration (µg C L^−1^ d^−1^)	135.9 ± 106.1 ^a^	117.8 ± 51.2 ^b^	118.7 ± 73.5 ^b^	117.0 ± 47.4 ^b^
Bacterial growth efficiency (%)	19.6 ± 16.1 ^a^	14.9 ± 5.4 ^b^	14.9 ± 9.5 ^b^	11.2 ± 7.2 ^c^
Bacterial carbon demand (µg C L^−1^ d^−1^)	158.8 ± 104.9 ^a^	140.0 ± 65.1 ^b^	135.0 ± 75.4 ^b^	130.5 ± 46.5 ^b^
Community respiration (µg C L^−1^ d^−1^)	317.1 ± 32.6 ^a^	290.7 ± 49.5 ^b^	198.0 ± 46.8 ^c^	158.0 ± 6.1 ^d^
Viral production (x 10^6^ VLP mL^−1^ d^−1^)	3.2 ± 2.2 ^a^	3.3 ± 1.5 ^a^	2.0 ± 1.1 ^b^	2.2 ± 0.5 ^b^
Viral mediated bacterial mortality (%)	19.7 ± 14.9 ^a^	11.7 ± 3.1 ^b^	13.1 ± 5.0 ^b^	22.9 ± 11.0 ^a^
Heterotrophic nanoflagellate (10^3^ cells mL^−1^)	6.4 ± 1.6 ^a^	5.1 ± 0.9 ^a^	6.3 ± 3.0 ^a^	3.8 ± 1.2 ^b^
Potential nanoflagellate grazing (10^9^ cells L^−1^ d^−1^)	1.8 ± 0.9 ^a^	2.3 ± 0.9 ^a^	1.2 ± 0.4 ^b^	0.6 ± 0.1 ^c^

**Table 3:** Pearson’s correlation coefficient (r) between different variables in the surface waters of Lake Saint-Gervais (*n* = 24).

	Temp	DOC	TDN	PO4	Chl	VA	BA	HNF	BP	BR	BGE	VP	Lysis
DOC	0.85 ***												
TDN	−0.49 *	−0.62 ***											
PO4	NS	NS	NS										
Chl	−0.72 ***	−0.55 **	NS	NS									
VA	0.77 ***	0.68 ***	NS	NS	−0.65 ***								
BA	0.77 ***	0.72 ***	NS	NS	−0.58 **	0.73 ***							
HNF	NS	NS	−0.53 **	NS	NS	NS	NS						
BP	0.52 **	0.55 **	NS	NS	NS	0.65 ***	0.63 ***	NS					
BR	NS	NS	NS	NS	NS	NS	NS	0.54 **	NS				
BGE	NS	NS	NS	NS	NS	NS	0.45 **	−0.45 **	0.64 ***	−0.50 **			
VP	0.49 *	0.54 **	NS	NS	NS	0.68 ***	0.52 *	NS	0.57 **	0.51 **	NS		
Lysis	NS	NS	NS	NS	NS	NS	−0.50 ***	NS	NS	0.71 ***	−0.55 **	NS	
FLG	0.64 ***	0.58 **	−0.41 *	NS	−0.42 *	0.67 ***	0.73 ***	NS	0.50 **	NS	NS	0.71 ***	0.49 **

Temp: water temperature, DOC: dissolved organic carbon, TDN: total dissolved nitrogen, PO4: orthophosphate, Chl: chlorophyll *a*, VA: viral abundance, BA: bacterial abundance, HNF: heterotrophic nanoflagellate abundance, BP: bacterial production, BR: bacterial respiration, BGE: bacterial growth efficiency, VP: viral production, FLG: potential flagellate grazing. Level of significance: * *p* < 0.05, ** *p* < 0.01, *** *p* < 0.001, NS: not significant.

## Data Availability

The original contributions presented in the study are included in the article/Supplementary Material, further inquiries can be directed to the corresponding author.

## Supplementary Materials

The following supporting information can be downloaded at: https://www.mdpi.com/article/10.3390/microorganisms12102061/s1, Figure S1. Map showing location of the study site in the French Massif Central region. Figure S2. Seasonal variation in water temperature and dissolved organic carbon concentration in the pelagic zone of Lake Saint Gervais. The data presented represent the averages of triplicate measurements. Figure S3. Regression plot showing the relationship between high nucleic acid bacteria (HNA) and bacterial production in Lake Saint-Gervais (y = 0.58x + 0.55, r = 0.75, *p* < 0.001, *n* = 24).
