# Supplementary material for: Impact of Top-Down Regulation on the Growth Efficiency of Freshwater Bacterioplankton
Source: Microorganisms. 2024 Oct 15;12(10):2061. doi: 10.3390/microorganisms12102061 (PMC11509854; doi:10.3390/microorganisms12102061)

Supplementary Figure S1. Map showing location of the study site in the French Massif Central region.

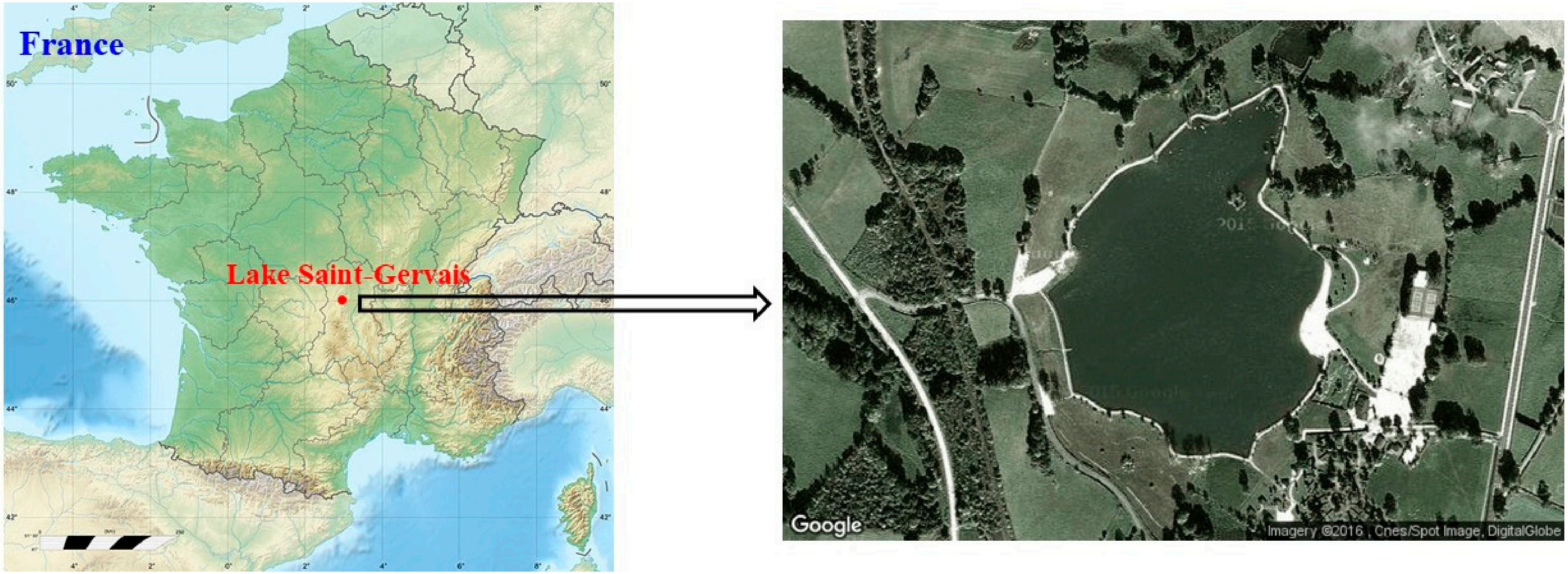

Supplementary Figure S2. Seasonal variation in water temperature and dissolved organic carbon concentration in the pelagic zone of Lake Saint Gervais. The data presented represent the averages of triplicate measurements.

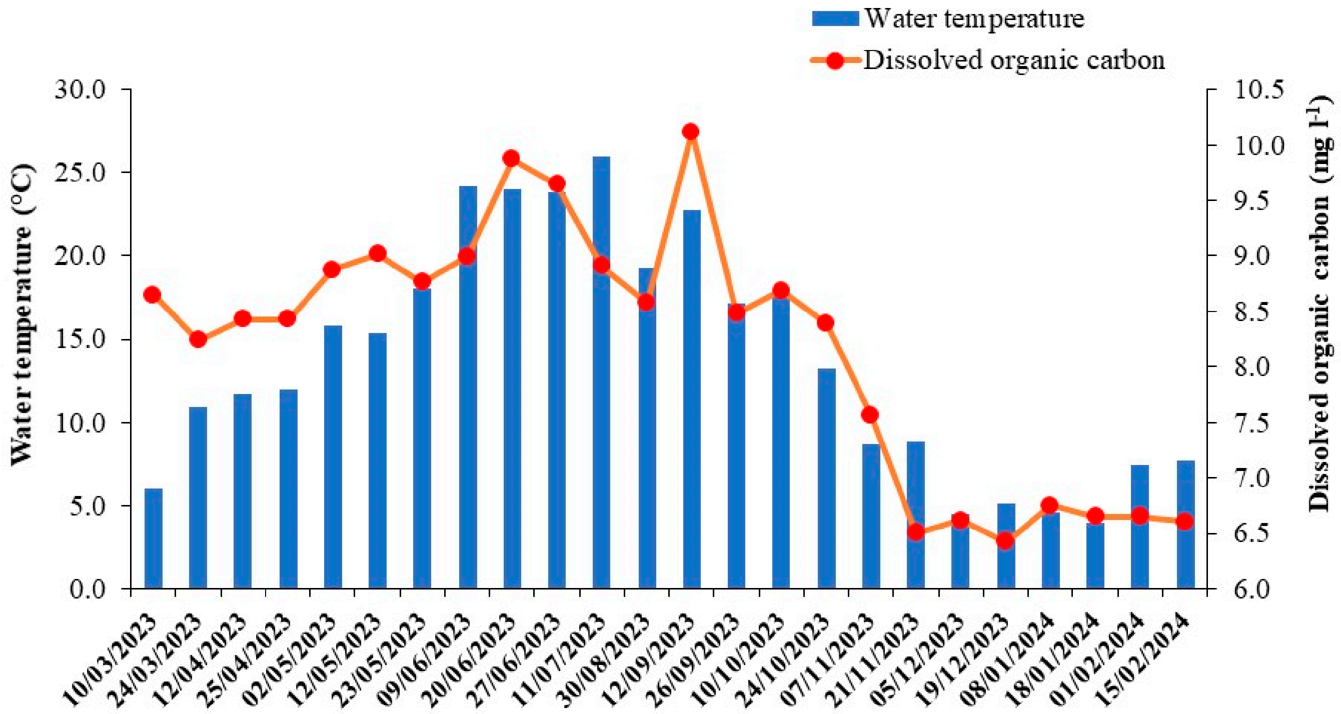

Supplementary Figure S3. Regression plot showing the relationship between high nucleic acid bacteria (HNA) and bacterial production in Lake Saint-Gervais ($y = 0.58x + 0.55$, $r = 0.75$, $p < 0.001$, $n = 24$).

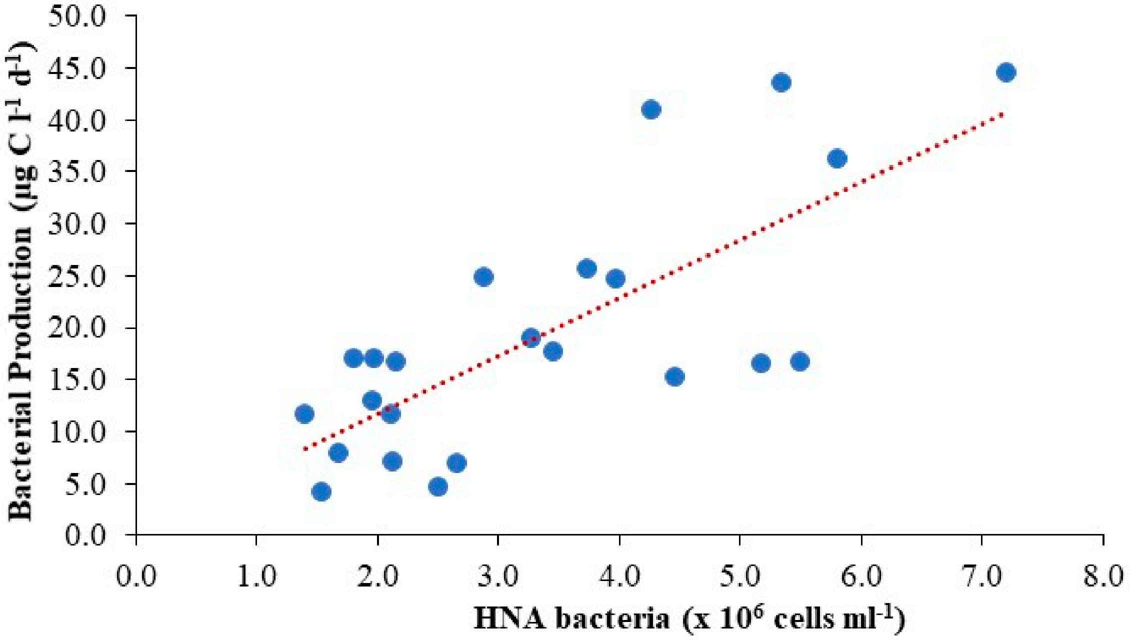

Supplement: Supplementary file 1 [file microorganisms-12-02061-s001.zip › microorganisms-3244071-supplementary.pdf]
